# Limited value of routine follow-up visits in chronic lymphocytic leukemia managed initially by watch and wait: A North Denmark population-based study

**DOI:** 10.1371/journal.pone.0208180

**Published:** 2018-12-27

**Authors:** Caroline Holm Nørgaard, Nikoline Buus Søgaard, Jorne Lionel Biccler, Laura Pilgaard, Mathias Holmsgaard Eskesen, Thordis Helga Kjartansdottir, Martin Bøgsted, Tarec Christoffer El-Galaly

**Affiliations:** 1 Department of Haematology, Aalborg University Hospital, Aalborg, Denmark; 2 Unit of Epidemiology and Biostatistics, Aalborg University Hospital, Aalborg, Denmark; 3 Department of Clinical Medicine, Aalborg University, Aalborg, Denmark; 4 Clinical Cancer Research Center, Aalborg University Hospital, Aalborg, Denmark; Donald and Barbara Zucker School of Medicine at Hofstra/Northwell, UNITED STATES

## Abstract

**Introduction:**

The majority of newly diagnosed chronic lymphocytic leukemia (CLL) patients are followed initially by watch and wait (WAW). Clinical practice varies and the value of frequent follow-up visits remains unclear. Thus, in this study we investigated the clinical value of follow-up visits for patients with CLL.

**Methods:**

We collected data from diagnosis and follow-up visits for patients diagnosed with CLL and managed by WAW in the North Denmark Region between 2007–2014. High- and low-risk group patients were determined by Binet stage, IgVH status, and cytogenetics at diagnosis. The effect of risk group allocation on the probability of receiving CLL-directed treatment within two years was included in a multivariable logistic regression model adjusted for age and blood test results.

**Results:**

273 patients were included in the study with a median follow-up of 3 years (IQR: 1.6–5.4). Overall, the median interval between follow-up visits was 98 days (95% CI: 96–100) (high-risk patients: 91 days [95% CI: 86–95] vs. low-risk patients: 105 days [95% CI: 100–110]). Among 2,312 follow-up visits, only 387 (17%) were associated with interventions. At the following time points: 6 months, 1 year, and 1.5 years, patients with low-risk CLL had significantly lower odds of initiating treatment compared to patients with high-risk CLL.

**Conclusion:**

WAW plays an important role in managing CLL. Interventions at follow-up visits were infrequent and low-risk patients had significantly lower risk of treatment initiation. We question the value of routine follow-up in CLL in the absence of changes in symptoms and/or blood test results.

## Introduction

Chronic lymphocytic leukemia (CLL) is the most common leukemia in the Western world and often characterized by an indolent clinical course [1]. More than 80% of patients are incidentally diagnosed by routine blood tests, and the majority present with early-stage disease upon diagnosis [2, 3]. Upfront treatment is not associated with better outcome for patients with asymptomatic, early-stage CLL and therefore, watch and wait (WAW) is a widely accepted initial management strategy [4–6].

A substantial fraction of CLL patients (up to 40%) managed by WAW will not require CLL-directed treatment during their lifetime [7]. Risk stratification using clinical staging, cytogenetics, and immunoglobulin heavy chain gene mutational (IgVH) status can identify patient subgroups at greater risk of developing symptomatic disease [7, 8]. Still, considerable heterogeneity within identified risk groups exists and many patients are offered lifelong follow-up in specialized hematology/oncology clinics.

The value of routine follow-up in CLL, however, is largely unknown, and local practice varies, as evidence-based follow-up guidelines do not exist.

Due to the ageing populations in the Western world and greater awareness of early signs of CLL, the number of CLL patients that will be managed by WAW is likely to increase over the coming years [7]. Therefore, studies that explore the value of multiple routine follow-ups in CLL are important, not only from a patient perspective, but also to ensure sustainable clinical practice in future health care. In the present study, we examined the findings and outcomes of routine follow-up visits for CLL.

## Methods

### Study cohort

Patients diagnosed with CLL in the North Denmark Region were identified using the local registrations in the Danish National Chronic Lymphocytic Leukemia Registry [9]. All patients who underwent diagnostic workup in the period May 2007–December 2014 and follow-up at the Department of Hematology at Aalborg University Hospital were screened for eligibility. Inclusion criterion for this retrospective study was initial management by WAW that lasted for at least 90 days. This study was approved by the Danish Data Protection Agency (ID: 2015–129) and the head of the Department of Hematology at Aalborg University Hospital. All analyses were performed on anonymized data.

### Data collection and variables

Baseline clinicopathologic characteristics were obtained from the local CLL Registry and health records. Follow-up visits were defined as any outpatient hospital visit to the Department of Hematology between diagnosis and initiation of CLL-directed treatment. Information from each visit were collected and included clinical findings (symptoms and physical examination), laboratory findings, and interventions.

Recorded symptoms included fatigue, weight loss, night sweats, subfebrilia/febrilia, infection tendency, bleeding tendency, and/or other possible CLL-related symptoms, such as loss of appetite, pain/fullness in the stomach, or anemia-related symptoms. Physical examination findings included lymph node or organ (spleen and liver) enlargement.

Laboratory results included hemoglobin levels, thrombocyte counts, leukocyte counts, and lactate dehydrogenase (LDH) levels. Leukocyte count was used as a surrogate for lymphocyte count, as leukocyte differential count was not routinely measured. Cut-offs for abnormal blood test results were defined to reflect considerable deviations of blood levels relevant for CLL: Hemoglobin < 6.2 mmol/L (< 10.0 g/dL)[10], thrombocytes < 100 x 10^9^/L (< 100,000/mm^3^) [10], leukocytes ≥ 30x10^9^/L (> 3,000/mm^3^) [11], and LDH ≥ 205 U/L for patients < 70 years and ≥ 255 U/L for patients ≥ 70 years. Presence of one or more of these values was defined as having abnormal blood test results.

An intervention at a follow-up visit was defined as follows: referral for medical imaging (X-ray, ultrasound, or CT-scan), ordering of biopsy (lymph node or bone marrow), request for additional/increased frequency of blood tests and/or follow-up visits, or treatment for CLL-complications (corticoid steroids for immunological complications such as hemolysis, blood transfusion, erythropoietin, or immunoglobulin substitution) during the follow-up period.

Patients were followed until they were started on CLL-directed treatment, discharged to primary care, death, whichever occurred first, or censored at December 31st, 2015.

Each patient was allocated to either a high- or low-risk CLL group. High-risk CLL was defined by the presence of one or more of the following risk factors: Binet B/C, 11q^-^/17p^-^, and/or unmutated IgVH, whereas low-risk CLL was defined by the absence of all of these clinicopathologic features (i.e. Binet A, no aberrations and/or 13q^-^ and/or Tri12, and mutated IgVH).

### Statistical analysis

Blood values were analyzed as continuous variables, while all other variables were categorical. Baseline characteristics were reported using median or mean for continuous variables and frequencies for categorical variables. Information on symptoms was available for all visits, while information about physical examinations was available from 79% of the visits. Blood tests were taken before 99% of visits.

The median follow-up time was estimated by reversing the role of events and censoring in the Kaplan-Meier estimator (the reverse Kaplan-Meier method) [12]. Time to first treatment was defined as the time from diagnosis to initiation of CLL-directed treatment and the cumulative incidence of initiating treatment was calculated by considering death as a competing risk.

Multivariable logistic regression models were used to inspect the effect of risk group allocation on the probability of receiving CLL-directed treatment within two years. Three different models, all adjusted for age and blood test results, were fitted and obtained by considering patients at risk, i.e. those who were not censored, alive, and had not yet received treatment, at the following time points: 6 months, 1 year, and 1.5 years post-diagnosis. The outcome variable in the logistic regression model was receiving treatment within two years after the given time points. The treatment status two years after the time point was missing for patients who experienced a competing risk, i.e. death, or were censored at that time. To account for the censoring and the competing risk, pseudo-values representing the probability that a subject received treatment within two years were used as the outcome in the multivariable logistic regression models [13]. Blood test results were collected in relation to the most recent follow-up visit before the three given time points (S1 Fig). The respective blood test values and age were rescaled so that, at baseline, their standard deviation equaled one

Median time between visits was calculated using the non-parametric Wang-Chang estimator and confidence intervals were obtained by bootstrapping [13, 14].

The effect of the clinical and laboratory findings on interventions was inspected using an Andersen-Gill model of the intensity, i.e. the event rate at a given time given the information available at that time, in which the findings were included as time-varying covariates, while standard errors were obtained using a robust jackknife estimator [15]. All statistical analyses were carried out using R version 3.4.1 (S1 Appendix).

## Results

### Study cohort

A total of 305 patients with newly diagnosed CLL were identified during the surveyed period (2007–2014). 32 patients were excluded due to treatment-initiation within 90 days of diagnosis, which left a total of 273 (90%) patients, initially managed by WAW, for further analyses (Table 1). Median age at diagnosis was 71 years and approximately 60% of patients were male. The cohort consisted of 66% patients with low-risk CLL and 34% with high-risk CLL.

**Table 1:** Baseline characteristics of the study participants.

**Age–yr**	
Median (range)	71 (33–98)
**Sex–*n* (%)**	
Male	164 (60.1)
**Hemoglobin < 6.2 mmol/L–*n* (%)**	
	10 (3.7)
**Thrombocytes <100 x 10**^**9**^**/L–*n* (%)**	
	4 (1.5)
**Leukocyte count ≥30 x 10**^**9**^**/L–*n* (%)**	
	70 (25.6)
**LDH ≥ 205 U/L for patients <70 years and ≥ 255 U/L for patients ≥70 years–*n* (%)**	
	39 (14.3)
**Beta2-microglobulin >340 nmol–*n* (%)**	
Yes	21 (7.7)
No	172 (63.0)
Unknown	80 (29.3)
**Binet stage–*n* (%)**	
A	240 (87.9)
B	30 (11.0)
C	3 (1.1)
**IgVH status–*n* (%)**	
Mutated IgVH	96 (35.2)
Unmutated IgVH	66 (24.2)
Unknown	111 (40.7)
**Cytogenetic status–*n* (%)**	
Normal	101 (37.0)
Aberrations	147 (53.8)
Del 13q14	120
Trisomy 12	33
Del 11q	14
Del17p	3
Unknown	25 (9.2)
**CD38 –*n* (%)**	
Yes	68 (24.9)
No	174 (63.7)
Unknown	31 (11.4)
**Performance status–*n* (%)**	
0	213 (78.0)
1	46 (16.9)
2	9 (3.3)
3	1 (0.4)
4	3 (1.1)
Unknown	1 (0.4)
**Risk group–*n* (%)**	
Low	181 (66.3)
High	92 (33.7)

### Characterization of follow-up period

The median follow-up time was 3 years (IQR: 1.6–5.4), and 2,312 follow-up visits were conducted during the study period, with median interval between visits being 98 days (95% CI: 96–100) (high-risk patients: 91 days [95% CI: 86–95] vs. low-risk patients: 105 days [95% CI: 100–110]). Within the first 2 years after diagnosis, the median time between visits was 98 days (95% CI: 98–98) (high-risk patients: 91 days [95% CI: 87–94] vs. low-risk patients 99 [95% CI: 92–103]). During the period starting from 2 years post-diagnosis the median time between visits was 182 days (95% CI: 170–195) (high-risk group: 126 [95% CI: 100–158] vs. low-risk group: 190 [95% CI: 185–196]).

By the end of the study, 126 patients (46%) remained on WAW at the hospital, 44 patients (16%) were discharged to primary care management due to stable disease and/or patient preferences, 76 patients (28%) had received CLL-directed treatment due to disease progression, and 27 patients (10%) had died (all-cause mortality) before initiation of CLL-directed treatment. Overall, 63 patients (23%) died during the study period.

### Risk group and initiation of CLL-directed treatment

**Fig 1:**
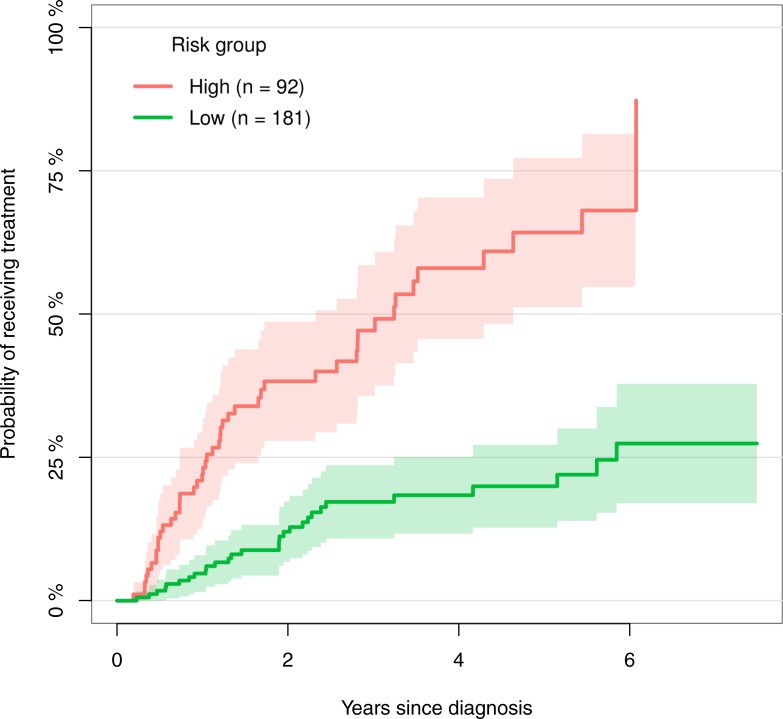
Cumulative incidence of the probability of initiating treatment. Cumulative incidence curves showing the probability of initiating treatment for low-risk patients (green) and high-risk patients (red).

The risk of initiating CLL-directed treatment within 5 years of diagnosis was 36% (95% CI: 29–44), with high-risk patients showing greater risk (70%, [95% CI: 56–84]), compared to low-risk patients (19%, [95% CI: 12–26], Fig 1).

**Fig 2:**
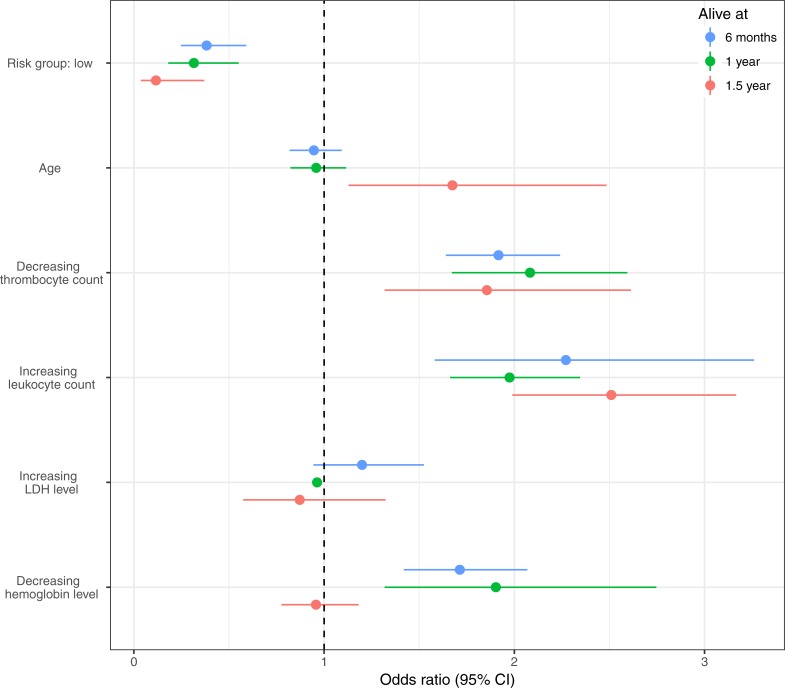
Odds ratio of 2-year predicted treatment initiation. Odds ratio for 2-year treatment initiation for patients at risk, estimated at the time points 6 months, 1 year, and 1.5 years post-diagnosis. The standardized thrombocyte count and hemoglobin level were multiplied by minus one in order to make the odds ratios correspond to a decrease in these counts.

Next, we investigated the effect of risk group on the 2-year odds of treatment initiation from the three selected time-points (6 months, 1 year, and 1.5 years). Odds ratios and 95% confidence intervals of the logistic regression models are shown in Fig 2. At each of the three selected time-points, the low-risk group was associated with decreased odds of 2-year treatment initiation compared to the high-risk group. Additionally, thrombocyte and leukocyte counts were, as expected, significantly associated with odds of 2-year treatment initiation at all three time points. Hemoglobin was significantly associated with odds of 2-year treatment initiation at 6 months and 1 year.

### Outcome of follow-up visits

Within the study period, 576 interventions occurred at 387 follow-up visits (17% of all visits). The probability of undergoing at least one intervention within 5 years of diagnosis was 74% (95% CI: 67–81). Analyzing time from diagnosis to first intervention, we found that 39% of patients had a first intervention during the first year of follow-up, 16% during the second year, 7% during the third year, and 16% between the fourth and sixth year after diagnosis.

Common interventions were *increase in the frequency of follow-up visits* (207 [9% of all follow-up visits]), *increase in the frequency of blood tests* (124 [5% of all follow-up visits]), *additional blood tests* (77 [3% of all follow-up visits]) and *imaging* (76 [3% of all follow-up visits]) (S1 Table).

Interventions at follow-up visits (387) were most frequently associated with the following constellations of clinical and laboratory findings: *symptoms and/or physical findings and abnormal blood test results* (210 [54%]), *abnormal test results* alone (87 [22%]), and *symptoms and/or physical findings* (54 [14%]), as shown in Table 2 (see S2 Table for the association between specific clinical and laboratory findings and interventions at follow-up visits). Altogether, *abnormal blood test results* were present at 297 (77%) follow-ups with interventions along with 69 (91%) cases of treatment initiations. *Physical examination alone* was related to 15 (4%) visits with interventions (in total physical examination alone was the reason for intervention in 0.6% of all visits (2312)) and was never the only documented reason for treatment initiation. The importance of *symptoms and/or physical findings and abnormal blood test results* (HR 9.8 [95% CI: 6.9–13.8]) as compared to *symptoms and/or physical findings* (HR 3.6 [2.4–5.4]) as well as *abnormal blood test results alone* (HR 3.1 [2.2–4.6]) was confirmed by their larger HR estimates.

**Table 2:** Association between clinical and laboratory findings and outcome of follow-up visits.

	**Number of follow-up visits with interventions**	Intensity of intervention*	**Number of follow-up visits with the decision to initiate CLL-directed treatment**
	**Frequency** ***n* (%)**	**HR (95% CI)**	**Frequency** ***n* (%)**
**Symptoms and/or physical findings**	54 (13.9)	3.6 (2.4–5.4)	5 (6.6)
Abnormal blood test results**	87 (22.5)	3.1 (2.2–4.6)	14 (18.4)
**Symptoms and/or** **Physical findings and Abnormal blood test results**	210 (54.3)	9.8 (6.9–13.8)	55 (72.4)
**Uncertain**	36 (9.3)	1 (REF)	2 (2.6)
**Total**	387 (100)		76 (100)

*Hazard ratios (HR) of an Andersen-Gill (AG) model for the intensity of the interventions with clinical and laboratory findings as time-varying covariates are reported.

**Hemoglobin < 6.2 mmol/L (< 10.0 g/dL)[10], thrombocytes < 100 x 10^9^/L (< 100,000/mm^3^) [10], leukocytes ≥30x10^9^/L (> 3,000/mm^3^) [11], and LDH ≥205 U/L for patients <70 years, or ≥255 U/L for patients ≥70 years.

## Discussion

This study examined the outcome of routine follow-up in CLL. Overall, we observed infrequent occurrence of interventions at follow-up visits. Furthermore, risk group allocation was significantly associated with 2-year odds of treatment during the first 1.5 years after diagnosis.

Prognostic modeling increasingly involves the use of genetic markers [8, 16]. This is useful for baseline risk stratification of newly diagnosed patients, as full information is available at that time. However, some patients with low-risk disease require treatment after a short WAW period and not all patients with high-risk CLL experience early progressions. Therefore, disease surveillance is commonly offered to all patients regardless of baseline risk stratification. Disease surveillance during WAW still relies on simple clinical and laboratory findings. The main purpose is to detect disease progression that requires interventions in the form of additional visits or tests, or initiation of CLL-directed treatment. Considering this, we decided to investigate whether clinical and laboratory findings could provide valuable information during WAW follow-up.

In the analyzed patient cohort, hemoglobin < 6.2 mmol/L and thrombocytes < 100 x 10^9^/L were rare finding during follow-up, which was expected both represent thresholds commonly used for treatment initiation [6].

The majority of CLL patients are managed by WAW for long periods of time and surveillance of a cancer disease without undergoing treatment can be a source of profound emotional distress and anxiety. Especially, emotional quality of life has been shown to be markedly impacted in CLL at all stages [17]. Moreover, emotional and social quality of life as well as levels of depression and anxiety are all very similar between CLL patients managed by WAW and those undergoing active CLL-directed treatment [18]. With this in mind, it would be valuable to obtain more dynamic estimations of disease progression risk during WAW to help reassure patients and potentially reduce worry and anxiety [7].

This study showed that both high- and low-risk CLL patients were followed closely during the follow-up period, with no significant difference between groups within the first two years (3.7 follow-up visits per year). Limited evidence exists on the frequency of follow-up during WAW. Shanafelt et al. have suggested the use of an individualized follow-up approach for Rai stage 0/Binet stage A, using molecular markers to stratify patients into risk groups [19]. Eichhorst et al. have suggested follow-up visits for early stage asymptomatic patients every 3–12 months [20], whereas Oscier et al. recommended WAW follow-up visits at least twice within the first year of diagnosis, and once per year after that [2]. Compared to these recommendations, low-risk patients in our cohort were seen twice yearly after two years of follow-up, which however was significantly less frequent compared to high-risk patients (seen roughly 3 times a year). Despite frequent follow-up visits in our study, the majority of visits (83%) did not result in any interventions and it is likely that more individualized follow-up schedules based on dynamic risk estimations could reduce the relatively high frequency of visits, without compromising timely intervention. The CLL International Prognostic Index (CLL-IPI) represents a novel and readily implementable tool to achieve more individualized WAW follow-up [8]. This prognostic score can identify four prognostic subgroups and although originally generated to predict overall survival, the index has been validated to predict time to first treatment in early stage CLL [21]. The most recent Danish CLL guidelines propose that the CLL-IPI should partially replace the current risk stratification used in this study, and thus future evaluation may prove the CLL-IPI a refined approach to more effectively risk stratify the large group of patients with early stage CLL [22].

Blood test results could further pave the way for more dynamic risk estimations to assess the need for follow-up visits at a given time. Moreover, visits to the outpatient clinic may in some cases be replaced by telemedicine if patients have no symptoms and blood tests do not indicate significant and unexpected disease progression. Telemedicine in cancer follow-up was reviewed by Dickinson et al. and did not appear to compromise patient satisfaction nor safety [23]. Moreover, in a pilot study by Overend et al., patients with indolent chronic hematologic malignancies (40% with CLL) were managed effectively and safely via a Teleclinic and 62% of patients preferred this over attending traditional outpatient clinics [24]. To that end, it is important to evaluate if telemedicine solutions as a replacement of outpatient visits reduce health anxiety among CLL patients with indolent disease. Other points to explore, are whether telephone/video follow-up visits could be nurse-led, potentially leading to a physician-led telephone/video visit or even a hospital visit in the presence of any concerns or symptoms. More follow-up in primary care may also be an option for some patients, possibly as a shared care solution.

It is important to note that this study relied on data not obtained for this particular study. Due to the retrospective nature of this study, it was not possible to assess how various factors weighed in on the physician’s decision to request interventions and treatment. In addition, by relying on symptoms/findings noted to clinical files we cannot exclude biased results such as minor symptoms not reported by the patients or interpreted by the physician.

In summary, WAW remains a cornerstone in managing CLL. We found that the majority of follow-up visits did not result in any interventions and that low-risk patients had significantly lower risk of initiating CLL-directed treatment compared to high-risk group patients. Furthermore, interventions and treatment initiation were found to be highly associated with abnormal blood test results and reported symptoms and may guide the need to come to the outpatient clinic.

## Supporting information

S1 Appendix R documentation. (PDF)

S1 Table Frequency of interventions at follow-up visits. (PDF)

S2 Table Association between disease-specific findings and interventions at follow-up visits. (PDF)

S1 Fig Use of blood test results in the treatment prediction model. (PDF)
