# Supplementary material for: Limited value of routine follow-up visits in chronic lymphocytic leukemia managed initially by watch and wait: A North Denmark population-based study
Source: PLoS One. 2018 Dec 27;13(12):e0208180. doi: 10.1371/journal.pone.0208180 (PMC6307783; doi:10.1371/journal.pone.0208180)
Supplement: S1 Appendix — (PDF) [file pone.0208180.s001.pdf]

```
#Require packages from library
packagesToLoad <- c("readxl", "dplyr", "DBI", "msm",
                    "prodlim", "survival", "lubridate",
                    "cmprsk", "MASS", "WriteXLS", "data.table",
                    "dtplyr", "car", "riskRegression", "lqmm",
                    "stats", "rpart", "geepack", "geepack",
                    "pseudo", "splines", "survrec")
lapply(packagesToLoad, library, character.only = T)

#################
## DATA FORMAT ##
#################

## Baseline: a data-frame which has the values of different variables measured
at baseline
## each row corresponds to a patient
##    timeTillFirstInt: the time from diagnosis till the first intervention
(measured in years)
##    status: status variable of timeTillFirstInt (censored = 0, dead = 1,
treatment start = 2, intervention = 3)
##      riskgroup: variable indicating the risk group
##      terminalTime: time from diagnosis till start of treatment or death
(measured in years)
##      failure: status variable for terminalTime (censored = 0, dead = 1,
treatment start = 2)
##      ldh: measured ldh value
##      haemoglobi: measured haemaglobin value
##      trombocyt: measured thrombodyte count
##      leukocyt: measured leukocyte count
##      age: age at diagnosis (measured in years)
##      patient_id: id of the patient


## Followup: a data-frame in which each row corresponds with a specific visit
##      patient_id: id of the patient
##      daysSinceDiagnosis: days between the diagnosis date and the visit
##      ldh: measured ldh value
##      haemoglobi: measured haemaglobin value
##      trombocyt: measured thrombodyte count
##      leukocyt: measured leukocyte count
##      terminalTime: time from diagnosis till start of treatment or death
(measured in years)
##      failure: status variable for terminalTime (censored = 0, dead = 1,
treatment start = 2)
##    symptoms: True if clinical symptoms were present at the visit
##      physical: True if physical findings were present at the visit
##    bloodEvent: True if abnormal blood values were present at the visit
##    intervention: True if an intervention was triggered due to this visit




###################################
## TIME TO INTERVENTION ANALYSES ##
###################################


##Creating model of time of first intervention, called "intDeathModel"
```

```
intDeathModel <- prodlim(Hist(timeTillFirstInt, status) ~ 1, data = baseline)

#Get the probability of receiving first treatment in xx year, "diff" returns
suitably lagged and iterated differences
diff(c(0, summary(intDeathModel, times = 1:6, cause = 3)$table$`3`[, 5]))

#Generate the figure depicting the probability of having > 0 interventions
plot(intDeathModel, cause = 3,  xlab = "Years since diagnosis", atrisk = F, ylab
= "Probability of having > 0 interventions")

#Probability of having at least 1 intervention 5-years post-diagnosis
summary(intDeathModel, times = 5)

##Creating model of time of first intervention, called "intDeathModelStrat",
stratified by risk group
intDeathModelStrat <- prodlim(Hist(timeTillFirstInt, status) ~ riskgroup, data =
baseline)

#Generate the figure depicting the probability of having > 0 interventions,
stratified by risk group
plot(intDeathModelStrat, cause = 3,  xlab = "Years since diagnosis",
     ylab = "Probability of having > 0 interventions",
     legend.title = "risk group", atrisk = F,
     legend.x = 0, legend.y = 1, legend.cex = 1)

#Probability of having at least 1 intervention 5-years post-diagnosis,
stratified by risk-group
summary(intDeathModelStrat, times = 5, cause = 3)

##Get the probability of receiving first treatment within xx years for the risk
groups
diff(c(0, summary(intDeathModelStrat, times = 1:6, cause =
3)$table$`3`$`riskgroup=high`[, 5]))
diff(c(0, summary(intDeathModelStrat, times = 1:6, cause =
3)$table$`3`$`riskgroup=low`[, 5]))

##Gray's test for the difference, for risk group
with(baseline, cuminc(ftime = timeTillFirstInt, fstatus = status, group =
riskgroup))$Tests

#Time to treatment analyses
#Create a variable containing time till treatment, death, or censoring,
whichever came first
baseline$terminalTime <- baseline$terminalTime / 365.24


#Median follow-up time
quantile(prodlim(Hist(terminalTime, failure != 0) ~ 1, data = baseline,
reverse=TRUE)) #3.09

##Creating model of time until treatment, called "treatmentDeathModel"
treatmentDeathModel <- prodlim(Hist(terminalTime, failure) ~ 1, data =
baseline)

#Generate the figure depicting the probability of receiving treatment for all
patients (Figure 1)
plot(treatmentDeathModel, cause = 2, atrisk = F,
     xlab = "Years since diagnosis",
     ylab = "Probability of receiving treatment")
```

```
#Probability of having received treatment 5 years post-diagnosis
summary(treatmentDeathModel, times = 5, cause = 2)

#Generate the figure "treatRiskStrat" depicting the probability of receiving
treatment for patients stratified by risk group
treatmentDeathModelStrat <- prodlim(Hist(terminalTime, failure) ~ riskgroup,
data = baseline)
plot(treatmentDeathModelStrat, cause = 2,
     xlab = "Years since diagnosis",
     ylab = "Probability of receiving treatment",
     legend.title = "Risk group", atrisk = F,
     legend.legend = c(paste0("High (n = ", sum(baseline$riskgroup == "high"),
")"),
                       paste0("Low (n = ", sum(baseline$riskgroup == "low"),
")")),
     legend.x = 0, legend.y = 1, legend.cex = 1, col = c("#F8766D", "#00BA38"))

#Probability of having received treatment 5 years post-diagnosis, stratified by
risk group
summary(treatmentDeathModelStrat, times = 5, cause = 2)

#Gray's test for the difference, for risk group
cuminc(baseline$terminalTime, baseline$failure, group =
baseline$riskgroup)$Tests

#Check probability of intervention given that there was no death, intervention,
censoring, or treatment in the first 12 months
##Creating the dataframe "landmarkData" with the landmark condtions
landmarkCond <- 1
landmarkWindow <- 2
landmarkData <- baseline[(baseline$timeTillFirstInt > landmarkCond), ]

##Creating model for the receiving the first intervention over time called
"lmarkModelFirstInt", given the landmark condition is met
lmarkModelFirstInt <- prodlim(Hist(timeTillFirstInt, status) ~ 1, data =
landmarkData)

#Generate the figure depicting the probability of receiving having >0
interventins, given the landmark condition is met
plot(lmarkModelFirstInt, cause = 3,
     xlab = "Years since diagnosis",
     ylab = "Probability of having > 0 interventions",
     legend.title = "risk group", atrisk = F,
     legend.x = 0, legend.y = 1, legend.cex = 1)

#Probabilty of receiving a first itnervention 3 years post-diagnosis (i.e. 2
years post landmark)
summary(lmarkModelFirstInt, cause = 3, times = landmarkCond + landmarkWindow)

##Creating model for the receiving the first intervention over time called
"lmarkModelFirstIntStrat",
#given the landmark condition is met, stratified by risk group
lmarkModelFirstIntStrat <- prodlim(Hist(timeTillFirstInt, status) ~ riskgroup,
data = landmarkData)

#Generate the figure depicting the probability of receiving having >0
interventins,
#given the landmark condition is met, stratified by risk group
plot(lmarkModelFirstIntStrat, cause = 3, ylab = "Probability of having > 0
interventions",
```

```
     legend.title = "risk group", atrisk = F,
     xlab = "Years since diagnosis",
     legend.x = 0, legend.y = 1, legend.cex = 1)

#Probabilty of receiving a first intervention 3 years post-diagnosis (i.e. 2
years post landmark), stratified by risk group
summary(lmarkModelFirstIntStrat, times = landmarkCond + landmarkWindow, cause =
3)

#Thus the probability of an intervention occuring remains quite likely even
after reaching the landmark

##Creating model for the receiving treatment over time called
"landmarkModelTr",
#given the landmark condition is met
landmarkModelTr <- prodlim(Hist(terminalTime, failure) ~ 1, data =
landmarkData)

#Generate the figure depicting the probability of receiving treatment,
#given the landmark condition is met,
plot(landmarkModelTr, cause = 2, legend.title = "risk group", atrisk = F,
     legend.x = 0, legend.y = 1, ylab = "Probability of receiving treatment",
     xlab = "Years since diagnosis")

##Creating model for the receiving treatment over time called
"landmarkModelTrStrat",
#given the landmark condition is met, stratified by risk group
landmarkModelTrStrat <- prodlim(Hist(terminalTime, failure) ~ riskgroup, data =
landmarkData)

#Generate the figure depicting the probability of receiving treatment,
#given the landmark condition is met, stratified by risk group
plot(landmarkModelTrStrat, cause = 2, legend.title = "risk group", atrisk = F,
     legend.x = 0, legend.y = 1, ylab = "Probability of receiving treatment",
     xlab = "Years since diagnosis", legend.cex = 1)

#Probabilty of receiving treatment 3 years post-diagnosis (i.e. 2 years post
landmark), stratified by risk group
summary(landmarkModelTrStrat, times = landmarkCond + landmarkWindow, cause = 2)

###Different landmarking: no treatment, death, or censoring the first year post-
diagnosis
landmarkData2 <- baseline[(baseline$terminalTime > landmarkCond), ]
landmarkData2$atLeastOneInt <- factor(ifelse(landmarkData2$timeTillFirstInt < 1,
"yes", "no"))

##Creating model for the receiving treatment over time, called
"landmarkModelTr2",
#given the landmark condition is met
landmarkModelTr2 <- prodlim(Hist(terminalTime, failure) ~ 1, data =
landmarkData2)

#Generate the figure depicting the probability of receiving treatment over time,
#given the landmark condition is met
plot(landmarkModelTr2, cause = 2, legend.title = "Intervention in\nthe first
year",
     atrisk = F, legend.x = 0, legend.y = 0.9, ylab = "Probability of receiving
treatment",
     xlab = "Years since diagnosis", legend.cex = 1)
```

```
##Creating model for receiving treatment called "landmarkModelTrStrat2",
#given the landmark condition is met
#+ stratified by whether or not the patients received at least one intervention
in the first year
landmarkModelTrStrat2 <- prodlim(Hist(terminalTime, failure) ~ atLeastOneInt,
data = landmarkData2)
x <- summary(landmarkModelTrStrat2, times = landmarkCond + landmarkWindow)
x

varDif <- x$table$`2`$`atLeastOneInt=yes`[1, 6]^ 2 +
x$table$`2`$`atLeastOneInt=no`[1, 6]^ 2
dif <- x$table$`2`$`atLeastOneInt=yes`[1, 5] - x$table$`2`$`atLeastOneInt=no`[1,
5]

2 * (1 - pnorm(abs(dif), sd = sqrt(varDif)))

#Generate the figure depicting the probability of receiving treatment over time,
#given the landmark condition is met
#+ stratified by whether or not the patients received at least one intervention
in the first year
plot(landmarkModelTrStrat2, cause = 2, legend.title = "Intervention in\nthe
first year",
     atrisk = F, legend.x = 0, legend.y = 0.9, ylab = "Probability of receiving
treatment",
     xlab = "Years since diagnosis", legend.cex = 1)

##Creating model for receiving treatment called "landmarkModelTrStrat2Risk",
#given the landmark condition is met
#+ stratified by whether or not the patients received at least one intervention
in the first year
#+ stratified by risk group
landmarkModelTrStrat2Risk <- prodlim(Hist(terminalTime, failure) ~
atLeastOneInt + riskgroup, data = landmarkData2)

#Generate the figure depicting the probability of receiving treatment over time,
#given the landmark condition is met
#+ stratified by whether or not the patients received at least one intervention
in the first year
#+ stratified by risk group
plot(landmarkModelTrStrat2Risk, cause = 2,
     atrisk = F, legend.x = 0, legend.y = 1, ylab = "Probability of receiving
treatment",
     xlab = "Years since diagnosis", legend.cex = 0.6)

#Adjust for risk-group using a stratified test
cuminc(landmarkData2$terminalTime, landmarkData2$failure, strata =
landmarkData2$riskgroup, group = landmarkData2$atLeastOneInt)$Tests


#############################################
## LANDMARKED LOGISTIC REGRESSION ANALYSES ##
#############################################

##Only use the observations with complete data on the necessary variables
baseline <- baseline[complete.cases(baseline[, c("ldh", "haemoglobi",
"trombocyt",  "leukocyt")]), ]
## restrict the analyses to patients who had their blood values available within
half a year post-diagnosis
baseline <- baseline[baseline$daysSinceDiagnosis < 365.24 / 2, ]
```

```
##Standardize the variables with respect to their sd at diagnosis, this makes
the visual display easier
bloodVar <- c("ldh", "haemoglobi", "trombocyt", "leukocyt")
for(selectedVar in bloodVar){
  selectedSD <- sd(baseline[, selectedVar])
  baseline[, selectedVar] <- baseline[, selectedVar] / selectedSD
  followup[, selectedVar] <- followup[, selectedVar] / selectedSD
}

ageSD <- sd(baseline[, "age"])
baseline[, "age"] <- baseline[, "age"] / ageSD
followup[, "age"] <- followup[, "age"] / ageSD

##Do the landmark analysis
landMark <- seq(0.5, 2, length.out = 31)
landWind <- 2

LMdata <- NULL

for (i in seq(along = landMark)){
  LM <- landMark[i] # current landmark time point
  datai <- baseline[baseline$terminalTime > LM, ] # select subjects at risk

  # pseudo-observations are calculated using pseudoci from pseudo package
  pseudoFit <- pseudoci(datai$terminalTime, datai$failure, tmax = landWind + LM)

  # get the latest blood values
  for(selectedPatient in datai$patient_id){
    patientData <- followup[followup$patient_id == selectedPatient, ]
    daysFromDiag <- patientData$daysSinceDiagnosis - LM * 365.24

    negativeDays <- daysFromDiag[daysFromDiag <= 0]

    for(selectedBlood in bloodVar){
      largestNegativeDay <- max(daysFromDiag[daysFromDiag < 0 &
!is.na(patientData[, selectedBlood])], na.rm = T)
      if(largestNegativeDay != - Inf){
        datai[datai$patient_id == selectedPatient, selectedBlood] <-
          patientData[which(daysFromDiag == largestNegativeDay)[1],
selectedBlood] # /
        # datai[datai$patient_id == selectedPatient, selectedBlood]
      }

    }
    datai[datai$patient_id == selectedPatient, "hadIntervention"] <-
      datai[datai$patient_id == selectedPatient, "timeTillFirstInt"] < LM
  }

  dfri <- data.frame(datai,
                     pseudov1 = pseudoFit$pseudo$cause1,
                     pseudov2 = pseudoFit$pseudo$cause2,
                     LM = rep(LM, nrow(datai)))
  LMdata <- rbind(LMdata, dfri)
}

##Create vectors to save the results in.
coefs <- NULL
varEst <- NULL

##Fit hte landmarked logistic regression models
```

```
for(i in landMark){
  datap <- LMdata[LMdata$LM == i, ]

  fit <- geese(pseudov2 ~ age + riskgroup + haemoglobi + ldh + trombocyt +
leukocyt
               , data = datap, id = patient_id,
               scale.fix = TRUE, family = gaussian, jack = TRUE, mean.link =
"logit",
               corstr = "independence", var = "binomial")
  coefs <- cbind(coefs, fit$beta)
  varEst <- cbind(varEst, diag(fit$vbeta))
}

##Plot
par(mfrow = c(2, 3))
for(i in 1:nrow(coefs)){
  ymax <- max(c(coefs[i, ], coefs[i, ] + 1.96 * varEst[i, ], coefs[i, ] - 1.96 *
varEst[i, ]))
  ymin <- min(c(coefs[i, ], coefs[i, ] + 1.96 * varEst[i, ], coefs[i, ] - 1.96 *
varEst[i, ]))
  plot(landMark, coefs[i, ], type = "l", main = rownames(coefs)[i], ylim =
c(ymin, ymax))
  lines(landMark, coefs[i, ] + 1.96 * varEst[i, ])
  lines(landMark, coefs[i, ] - 1.96 * varEst[i, ])
}

##Forest plot (Figure 2)
whichIndices <- which(landMark %in% c(0.5, 1, 1.5))
selectedLM <- c("6 months", "1 year", "1.5 year")

coefNames <- factor(c("Age", "Risk group: low", "Decreasing \n hemoglobin
level", "Increasing \n LDH level", "Decreasing \n thrombocyte count",
"Increasing \n leukocyte count"),
                    levels = rev(c("Risk group: low", "Age", "Decreasing \n
thrombocyte count", "Increasing \n leukocyte count",
                                   "Increasing \n LDH level", "Decreasing \n
hemoglobin level")))

coefs[c(4, 6), ] <- - coefs[c(4, 6), ]

forestPlotFrame <- data.frame(x = coefNames,
                              y = exp(as.numeric(coefs[-1, whichIndices])),
                              ymin = exp(as.numeric(coefs[-1, whichIndices] -
1.96 * varEst[-1, whichIndices])),
                              ymax = exp(as.numeric(coefs[-1, whichIndices] +
1.96 * varEst[-1, whichIndices])),
                              col = factor(rep(selectedLM, each = nrow(coefs) -
1),
                                           levels = rev(selectedLM)))

coefPlot <- ggplot(forestPlotFrame, aes(x = x, y = y, ymin = ymin, ymax = ymax,
col = as.factor(col))) +
  geom_pointrange(position =  position_dodge(width = 0.5)) +
  coord_flip() +  # flip coordinates (puts labels on y axis)
  ylab("Odds ratio (95% CI)") +
  theme_bw() +
  geom_hline(yintercept = 1, linetype = 2) +
  guides(colour = guide_legend(reverse = T, title = "Alive at")) +
  theme(legend.justification = c(1, 1),
        legend.position = c(1, 1),
```

```
        legend.background = element_rect(fill="transparent"),
        axis.title.y = element_blank(),
        plot.title = element_text(hjust = 0.5),
        axis.text.y = element_text(vjust = 0.5, hjust = 0.5))

coefPlot



#################################
## MEDIAN TIME BETWEEN VISITS ##
#################################


##Only select those visits corresponding to patients for which
##Blood values etc. were available at baseline
tempFollowup <- followup[followup$patient_id %in% baseline$patient_id, ]
tempFollowup <- tempFollowup[tempFollowup$daysSinceDiagnosis > 0, ]

##Create a data-set that contains the gap-times between visists, i.e. time from
previous visit to current visit
cause <- NULL
tstart <- NULL
tstop <- NULL
risk <- NULL
patientID <- NULL

for(selectedPatient in unique(tempFollowup$patient_id)){
  selectedData <- tempFollowup[tempFollowup$patient_id == selectedPatient, ]
  selectedData <- selectedData[order(selectedData$daysSinceDiagnosis), ]

  differences <- diff(c(0, selectedData$daysSinceDiagnosis))
  risk <- c(risk, rep(baseline[which(baseline$patient_id == selectedPatient),
"riskgroup"],
                      nrow(selectedData)))

  tempstart <- c(0, cumsum(differences))
  tstop <- c(tstop, tempstart[- 1])
  tstart <- c(tstart, tempstart[- length(tempstart)])

  patientTime <- selectedData$daysSinceDiagnosis
  patientID <- c(patientID, rep(selectedPatient, nrow(selectedData)))
  patientCause <- rep(1, nrow(selectedData))

  if((selectedData$failure == 1)[1]){
    patientCause[which(patientTime == max(patientTime))] <- 2
  } else if ((selectedData$failure == 2)[1]){
    patientCause[which(patientTime == max(patientTime))] <- 3
  } else {
    patientCause[which(patientTime == max(patientTime))] <- 0
  }
  cause <- c(cause, patientCause)
}

##Gap times between visists
timeVar <- tstop - tstart
visitFrame <- data.frame(patientID, timeVar, cause = cause == 1, risk)

##Create a data-set which focuses on the first two years post-diagnosis and
censores later events
```

```
selected <- tstart < 2 * 365.24
adjusted <- tstop >= 2 * 365.24
tempStop <- tstop
tempStop[adjusted] <- 2 * 365.24
tempCause <- cause
tempCause[adjusted] <- 0

visitFrame2 <- data.frame(timeVar = (tempStop - tstart)[selected],
                          patientID = patientID[selected],
                          cause = tempCause[selected] == 1,
                          risk = risk[selected])

##Create a data-frame containing visits happening more than two years post
diagnoasis
selected <- tstart > 2 * 365.24
visitFrame3 <- data.frame(timeVar = timeVar[selected],
                          patientID = patientID[selected],
                          cause = cause[selected] == 1,
                          risk = risk[selected])

##Make the risk group a factor in all three data-frames
visitFrame$risk <- factor(visitFrame$risk)
visitFrame2$risk <- factor(visitFrame2$risk)
visitFrame3$risk <- factor(visitFrame3$risk)

##Calculate the median time between visists by bootrapping a recurrent event
model
minmin <- function(y, xx) {
  if (any(!is.na(y) & y == 0.5)) {
    if (any(!is.na(y) & y < 0.5))
      0.5 * (min(xx[!is.na(y) & y == 0.5]) + min(xx[!is.na(y) &
                                                    y < 0.5]))
    else 0.5 * (min(xx[!is.na(y) & y == 0.5]) + max(xx[!is.na(y) &
                                                       y == 0.5]))
  }
  else min(xx[!is.na(y) & y <= 0.5])
}

bootResults <- function(covFormula, data, B){
  formulaSurvrec <- update.formula(covFormula, Survr(patientID, timeVar, cause)
~ .)

  fitOrig <- survfitr(formulaSurvrec, data = data, type = "wa")
  nStrata <- ifelse(is.null(attr(fitOrig, "strata")), 1, length(fitOrig))
  if(nStrata == 1){
    tempFun <- function(original, selectedPats) {
      fit <- survfitr(formulaSurvrec,
                      data = data[data$patientID %in% original[selectedPats], ],
                      type = "wa")
      minmin(fit$survfunc, fit$time)
    }
  } else {
    tempFun <- function(original, selectedPats) {
      fit <- survfitr(formulaSurvrec,
                      data = data[data$patientID %in% original[selectedPats], ],
                      type = "wa")
      bootEst <- NULL
      for(i in 1:nStrata){
        bootEst <- c(bootEst, minmin(fit[[i]]$survfunc, fit[[i]]$time))
      }
```

```
      bootEst
    }
  }
  bootObj <- boot(unique(data$patientID), tempFun, B)
  if(nStrata == 1){
    bootCI <- boot.ci(bootObj, type = "norm")
    print(paste("Rec model, median:", minmin(fitOrig$survfunc, fitOrig$time),
                "95CI (", bootCI$normal[1, 2], ";", bootCI$normal[1, 3], ")"))
  } else {
    for(i in 1:nStrata){
      bootCI <- boot.ci(bootObj, type = "norm", index = i)
      print(paste("Rec model, ", names(fit)[i],", median:",
                  minmin(fitOrig[[i]]$survfunc, fitOrig[[i]]$time),
                  "95CI (", bootCI$normal[1, 2], ";", bootCI$normal[1, 3], ")"))

    }
  }
}

##Get the results of the bootstrapping for the three data-frames
set.seed(87914)
bootResults(Survr(patientID, timeVar, cause) ~ 1, visitFrame, 999)
bootResults(Survr(patientID, timeVar, cause) ~ risk, visitFrame, 999)

bootResults(Survr(patientID, timeVar, cause) ~ 1, visitFrame2, 999)
bootResults(Survr(patientID, timeVar, cause) ~ risk, visitFrame2, 999)

bootResults(Survr(patientID, timeVar, cause) ~ 1, visitFrame3, 999)
bootResults(Survr(patientID, timeVar, cause) ~ risk, visitFrame3, 999)


########################################################################
## TIME-VARYING EFFECT MODELS FOR INTERVENTION AND TREATMENT TRIGGERS ##
########################################################################

##Reorganize the variables a bit
followup$symPhys <- (followup$symptoms | followup$physical) &
!followup$bloodEvent
followup$blood <- !(followup$symptoms | followup$physical) & followup$bloodEvent
followup$symPhysBlood <- (followup$symptoms | followup$physical) &
followup$bloodEvent

##Order data according to patient_id and withing patient according in order of
visit data
followup <- followup[order(followup$patient_id, followup$daysSinceDiagnosis), ]

##Create a tsart and tstop date corresponding to the time of the last visit and
current visit
followup$tstart <- NULL
followup$tstop <- followup$daysSinceDiagnosis

for(patientID in unique(followup$patient_id)){
  selected <- which(followup$patient_id == patientID)
  followup[selected, "tstart"] <- c(0, followup[selected[- length(selected)],
"daysSinceDiagnosis"])
}

##Create a counter of the number of previous visits
for(patientID in unique(followup$patient_id)){
  selected <- followup$patient_id == patientID
```

```
  followup[selected, "nVisits"] <- 0:(sum(selected) - 1)
}

##Fit a recurent event model with interventions as outcome with proportional
covariate effects (Table 2 )
interventionMod <- coxph(Surv(tstart, tstop, followup$intervention) ~
                           symPhys + blood + symPhysBlood,
                         method="breslow", robust=TRUE, data = followup)

round(exp(interventionMod$coefficients), 2)
round(exp(interventionMod$coefficients + 1.96 *
sqrt(diag(interventionMod$var))), 2)
round(exp(interventionMod$coefficients - 1.96 *
sqrt(diag(interventionMod$var))), 2)

##Fit a cox ph model with time-varying effects
treatmentMod <- coxph(Surv(tstart, tstop, followup$failure == 2) ~ blood +
symPhys + symPhysBlood,
                      method = "breslow", robust = TRUE, data = followup)
```
