# Supplementary material for: Limited value of routine follow-up visits in chronic lymphocytic leukemia managed initially by watch and wait: A North Denmark population-based study
Source: PLoS One. 2018 Dec 27;13(12):e0208180. doi: 10.1371/journal.pone.0208180 (PMC6307783; doi:10.1371/journal.pone.0208180)
Supplement: S1 Table — (PDF) [file pone.0208180.s002.pdf]

S1 Table: Frequency of interventions at follow-up visits

Intervention	Overall frequency
	n (%)
**X-ray**	10 (0.43)
**CT**	48 (2.07)
**Ultrasound**	18 (0.78)
**Biopsy**	33 (1.43)
**Additional blood tests**	77 (3.33)
**Increased blood test frequency**	124 (5.36)
**Increased visit frequency**	207 (8.95)
**Treatment for CLL-complications**	38 (1.64)
**Other**	21 (0.91)
**Sum**	576*

Overall frequency of interventions, including percentage of total number of follow-up visits (2312). *Note that more than one intervention could be made per follow-up visit.
