# Supplementary material for: Limited value of routine follow-up visits in chronic lymphocytic leukemia managed initially by watch and wait: A North Denmark population-based study
Source: PLoS One. 2018 Dec 27;13(12):e0208180. doi: 10.1371/journal.pone.0208180 (PMC6307783; doi:10.1371/journal.pone.0208180)
Supplement: S2 Table — (PDF) [file pone.0208180.s003.pdf]

S2 Table: Association between disease-specific findings and interventions at follow-up visits.

Clinical and laboratory findings	All	Low-risk group	High-risk group
	Frequency *n*	Frequency	Frequency
Symptoms			
Fatigue	95	50	45
Weight loss	24	9	15
Night sweats	53	15	38
Subfebrilia/febrilia	6	4	2
Infection tendency	57	29	28
Bleeding tendency	1	0	1
Other CLL relevant	34	21	13
Physical examination			
New finding of enlarged Lymph node/spleen/liver	131	60	71
Suspected progression of lymph node/spleen/liver enlargement	76	31	45
Other	1	1	0
Blood values			
Hemoglobin <6.2 mmol	35	25	10
Thrombocytes <100 mmol/L	29	19	10
Leukocyte count ≥30x10^9/L	226	107	119
LDH ≥205 U/L for patients <70 years and ≥255 U/L for patients ≥70 years	117	53	34

Overall frequency of disease-specific findings at follow-up visits with intervention occurrence (387) with the respective risk group distribution.
