# Supplementary material for: Limited value of routine follow-up visits in chronic lymphocytic leukemia managed initially by watch and wait: A North Denmark population-based study
Source: PLoS One. 2018 Dec 27;13(12):e0208180. doi: 10.1371/journal.pone.0208180 (PMC6307783; doi:10.1371/journal.pone.0208180)

S1 Fig. Use of blood test results in the treatment prediction model. Follow-up of CLL patients from diagnosis and the use of blood test results to predict odds of 2-year treatment initiation at the three given time points: 6 months, 1 year, and 1.5 years.

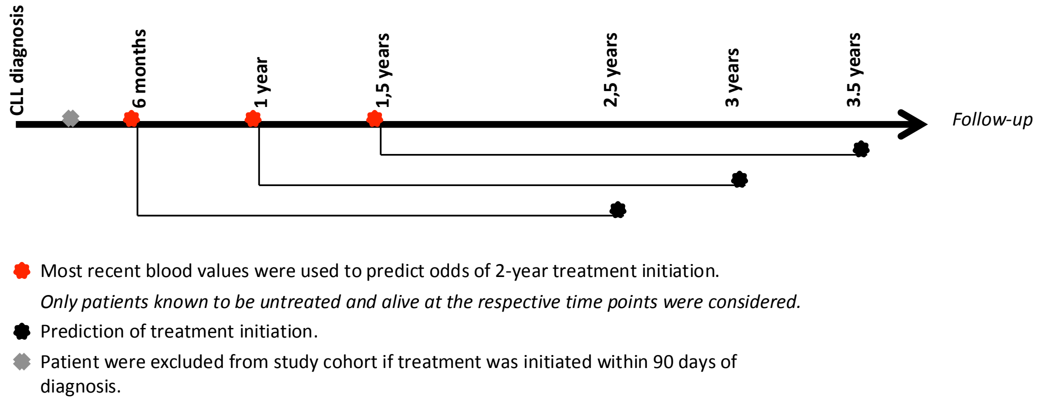

Supplement: S1 Fig — (PDF) [file pone.0208180.s004.pdf]
